# On the evaluation of the fidelity of supervised classifiers in the prediction of chimeric RNAs

**DOI:** 10.1186/s13040-016-0112-6

**Published:** 2016-11-02

**Authors:** Sacha Beaumeunier, Jérôme Audoux, Anthony Boureux, Florence Ruffle, Thérèse Commes, Nicolas Philippe, Ronnie Alves

**Affiliations:** 1 Institut de Médecine Régénératrice et de Biothérapie, INSERM U1183, CHU Montpellier, Montpellier, France; 2 Institut de Biologie Computationnelle, Université Montpellier, Montpellier, France; 3 Laboratoire d’Informatique, de Robotique et de Microélectronique de Montpellier, Université Montpellier, UMR 5506 CNRS, Montpellier, France; 4 PPGCC, Universidade Federal do Pará, Belém, Brazil; 5 Instituto Tecnológico Vale, Belém, Brazil

**Keywords:** Chimeric RNAs, Transcriptomics, Classification, Ensemble learning, Data simulation

## Abstract

**Background:**

High-throughput sequencing technology and bioinformatics have identified chimeric RNAs (chRNAs), raising the possibility of chRNAs expressing particularly in diseases can be used as potential biomarkers in both diagnosis and prognosis.

**Results:**

The task of discriminating true chRNAs from the false ones poses an interesting Machine Learning (ML) challenge. First of all, the sequencing data may contain false reads due to technical artifacts and during the analysis process, bioinformatics tools may generate false positives due to methodological biases. Moreover, if we succeed to have a proper set of observations (enough sequencing data) about true chRNAs, chances are that the devised model can not be able to generalize beyond it. Like any other machine learning problem, the first big issue is finding the good data to build models. As far as we were concerned, there is no common benchmark data available for chRNAs detection. The definition of a classification baseline is lacking in the related literature too. In this work we are moving towards benchmark data and an evaluation of the fidelity of supervised classifiers in the prediction of chRNAs.

**Conclusions:**

We proposed a modelization strategy that can be used to increase the tools performances in context of chRNA classification based on a simulated data generator, that permit to continuously integrate new complex chimeric events. The pipeline incorporated a genome mutation process and simulated RNA-seq data. The reads within distinct depth were aligned and analysed by CRAC that integrates genomic location and local coverage, allowing biological predictions at the read scale. Additionally, these reads were functionally annotated and aggregated to form chRNAs events, making it possible to evaluate ML methods (classifiers) performance in both levels of reads and events. Ensemble learning strategies demonstrated to be more robust to this classification problem, providing an average AUC performance of 95 % (ACC=94 %, Kappa=0.87 %). The resulting classification models were also tested on real RNA-seq data from a set of twenty-seven patients with acute myeloid leukemia (AML).

**Electronic supplementary material:**

The online version of this article (doi:10.1186/s13040-016-0112-6) contains supplementary material, which is available to authorized users.

## Background

A chimeric RNA (chRNA) is a RNA molecule that is made of two or more pieces of RNAs from different loci. Chimeric RNAs are formed from genetic rearrangements like translocation, inversion, deletion or copy number variation. Other studies have highlighted that many other chRNA could be also generated from post-transcriptional mechanisms such as read-through and splicing, transcription of short homologous sequence slippage or from trans-splicing [1].

High-throughput sequencing technology and bioinformatics have identified chRNA, raising the possibility of chRNAs expressing particularly in diseases can be used as potential biomarkers in both diagnosis and prognosis. However, only a limited number of chimeric transcripts and their associated protein products have been characterised to date, most of them resulting from chromosomal translocation and associated with cancer [2, 3]. For instance the well-known gene fusion in chronic myelogenous leukemia leading to an mRNA transcript that encompass the 5’ end of the *BCR* gene and the 3’ end of the *ABL* gene, producing the chRNA BCR–ABL.

Most protocols used to identify chimeric transcripts rely on a reverse transcription step and the reverse transcriptase is known to switch templates, thus creating chimeric artifacts in vitro [4]. Thus, it remains unclear what proportion of putative chimeric transcripts are true, and of these how many are translated. With the rapid evolution of genomic research, next generation sequencing (NGS) has become widely available. Sequences in databases or sequences directly from RNA-seq often contain information that leads to false results when they are analysed with bioinformatics approaches. This is partially due to the fact that the first stage of the available bioinformatic tools for finding chRNA basically rely on mapping, where reads are aligned with respect to the reference genome used by the algorithm. Additionally, the length and depth of sequencing might be inadequate for covering all exon junctions, and given that repeated sequences are present, clearly, the alignment of sequences to specific regions is highly biased to sequencing. Next to mapping, a set of filters based on several technological or biological assumptions are applied to reduce the set of putative chRNAs.

Many tools have been developed to detect chRNAs [5], though many complications arise when trying to provide a complete benchmark. Each one of these tools have particular constraints regarding installation, parametrization, usability, and database(s) or software(s) dependencies. Liu et al. [6] provided an overall evaluation of thirteen strategies, suggesting the combination of three of them as a robust solution. In fact, there is a trend to explore combination of algorithms to boost the analysis of chimera detection. However, a good combination of software requires proper criteria of selections based on metrics according to the biological question in order to choose the most suitable ones. In fact, the basic information shared in common by all these softwares is the initial sequenced reads which is distinctively explored by each of them. In other words, each software integrates its own algorithm and different external features and database to define proper margins or “rule(s)” to classify potential chRNAs. If one is able to make such abstraction (or rules) from the reads, it might be an interesting alternative to directly explore models generated from classifiers. In this work, we propose a different angle of discussion, model-oriented rather than comparing software for chRNAs detection which are not necessarily comparable because there are questions-oriented.

The task of discriminating true chRNA from the false ones poses an interesting Machine Learning (ML) challenge. First of all, the sequencing data is highly biased and thus predicting the real signal from the noise can be a hard task. Furthermore, even if we succeed to have a proper set of observations (enough sequencing data) about true chRNAs, chances are that the devised model can not be able to generalize beyond it.

Models generalization is the “holy grail” of machine learning. ML methods must be able to see beyond the data observed in order to generalize beyond it. The famous “no free lunch” theorem of Wolpert says that no learner can beat a random guessing over all possible functions to be learned. Ensemble strategies overcome the no-free-lunch dilemma by combining the outputs of many classifiers assuming that each classifier performs well in particular domains while being sub-optimal in others. Machine learning and ensemble based systems has been applied successfully in several data applications [7, 8]. The main idea behind the ensemble strategy is simple, “rely on the feedback of a bunch of specialists”. Thus, a combination of several models might better estimate the margins boundaries in the hypothesis space, reducing the bias and variance errors. Bias is a learner’s tendency to consistently learn the same wrong thing. Variance is the tendency to learn random things irrespective of the real signal [9]. Empirical and theoretical evidence show that some ensemble learning techniques like bagging act as a variance reduction mechanism, i.e., they reduce the variance component of the error. Boosting strategies reduce both the bias and variance parts of the error. It sounds that the bias error is usually reduced in the early iterations, while variance error decreases in later ones. Indeed, the key component behind ensemble success is the concept of classifier diversity [7]. Classifier diversity can be achieved in several ways. The most known approach is to use different training datasets to train individual classifiers. Such datasets are often obtained through resampling techniques, such as bootstrapping or bagging, where training data subsets are drawn randomly, usually with replacement, from the entire training data.

Most of the available gene fusion-finders methods [5, 10] do not explore the potentials of applying ML techniques to this classification problem. As far as we are aware of, only two gene fusion-finders, namely EricScript [11] and deFuse [12], have explored AdaBoost. AdaBoost generates a set of hypotheses and combines them through weighted majority voting of the classes predicted by the individual hypotheses. The hypotheses are generated by training a weak classifier, using instances drawn from an iteratively updated distribution of the training data. This distribution update ensures that instances misclassified by the previous classifier are more likely to be included in the training data of the next classifier. Hence, consecutive classifiers’ training data are geared towards increasingly hard-to-classify instances. As fas as we are concerned, there is no further literature in finding chRNAs using bagging-oriented strategies. Bagging is one of the pioneers in ensemble learning. Diversity in bagging is obtained by using bootstrapped replicas of the training data: different training data subsets are randomly drawn, with replacement, from the entire training data. Each training data subset is employed to train a distinct classifier of the same type. Individual classifiers are then combined by taking a majority vote of their decisions. For any given instance, the class chosen by most classifiers is the ensemble decision. Random Forest (RF) is a variation of the bagging algorithm, and it can be created from individual decision trees, whose certain training parameters vary randomly. Such parameters can be bootstrapped replicas of the training data, as in bagging, but they can also be different feature subsets as in random subspace methods. In this work we have successfully explored RF models in the prediction of chRNAs in both read and event levels.

Like any other machine learning problem, the first big issue is about the data [9]. As far as we are concerned, there is no common benchmark data available. Indeed, even the definition of a classification baseline is lacking in the related literature. In this work we are moving towards a benchmark data and a fair comparison analysis unraveling the role of supervised classifiers in finding chRNAs. All benchmark data and results are freely available at https://sites.google.com/site/alvesrco/chimeres.

## Methods

### Simulated data sets

This procedure modifies the sequence of the input reference genome by introducing random point mutations (SNV), insertions and deletions (or indels), as well as translocations. Substitutions and indels are introduced at random genomic locations at rates chosen by the user. By default, one every 1,000 nucleotides will be substituted (a rate of 0.1 %), while at 1/10,000 positions, an indel will be introduced (a rate of 0.01 %). At a substituted position, the new nucleotide is chosen at random with equal probability among the three other possibilities. For indels, the length is chosen uniformly within a range [1,15] nucleotides and the inserted sequence are chosen randomly. Regarding translocations, whose goal is to generate gene fusions, the exchanged chromosomal locations are chosen within annotated genes. The genome simulator takes a gene annotation file as input to get the positions of all exons. For a translocation, two exons of two different genes are chosen at random with an input probability, then we perform a bidirectional exchange of the 3 prim part of one gene with the 5prim part the other gene and the recripocal, thereby creating two chimeric, or fusion genes. Exons are never splitted by this process, as we hypothesized that fusion genes with disrupted exons are counterselected by evolution. For the sake of simplicity, the simulator generates chimeras using reads encoded on the forward strand only. Expressed chimeric RNAs will be covered by RNA-Seq reads; it is worth noting that both fused genes generated by a translocation are, as any other gene, not necessarily expressed, nor covered by reads. Another alternative for generating artificial gene fusions is by using the FUSIM tool [13], though the pipeline does not simulate RNA-seq data including chimeras, it only provides their associated sequences. The simulated data generator is packaged in a tools called SimCT (not yet published) that can easily integrate new complex chimeric events.

### Feature engineering

**Fig. 1:**
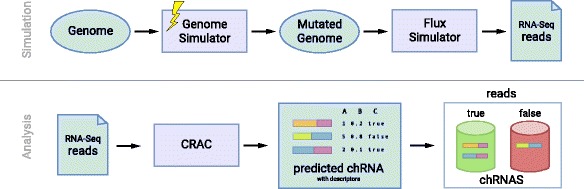
The benchmark pipeline covering mutations along the genome and simulation of RNA-seq data with Flux Simulator, followed by a chRNA labeling step with CRAC tool. CRAC’s algorithm is based on the study of *k*−*m* *e* *r* support and location profiles, being a valuable source of information to extract quantitative features for machine learning models

We have developed a benchmark pipeline (Fig. 1) using the above genome simulation procedure along with i) the generation of corresponding RNA-seq reads by Flux Simulator [14] and ii) labelling chRNAs classes through the use of CRAC Software [15].

CRAC has been chosen to perform this experimental study for two main reasons. First of all, it is a splice-aware mapping software that integrates the identification of chimeric junctions with a high confidence ratio. The second reason is that CRAC’s algorithm is based on the study of *k*−*m* *e* *r* support and location profiles which provides for every *k*−*m* *e* *r* of a read its number of occurrences in the whole read collection and all its possible locations on the reference genome. These profiles are of an extremely valuable source of information to extract quantitative features that define chimeric junctions at the read “level” and therefore, suitable for the development of ML models [see Additional file 1].

**Fig. 2:**
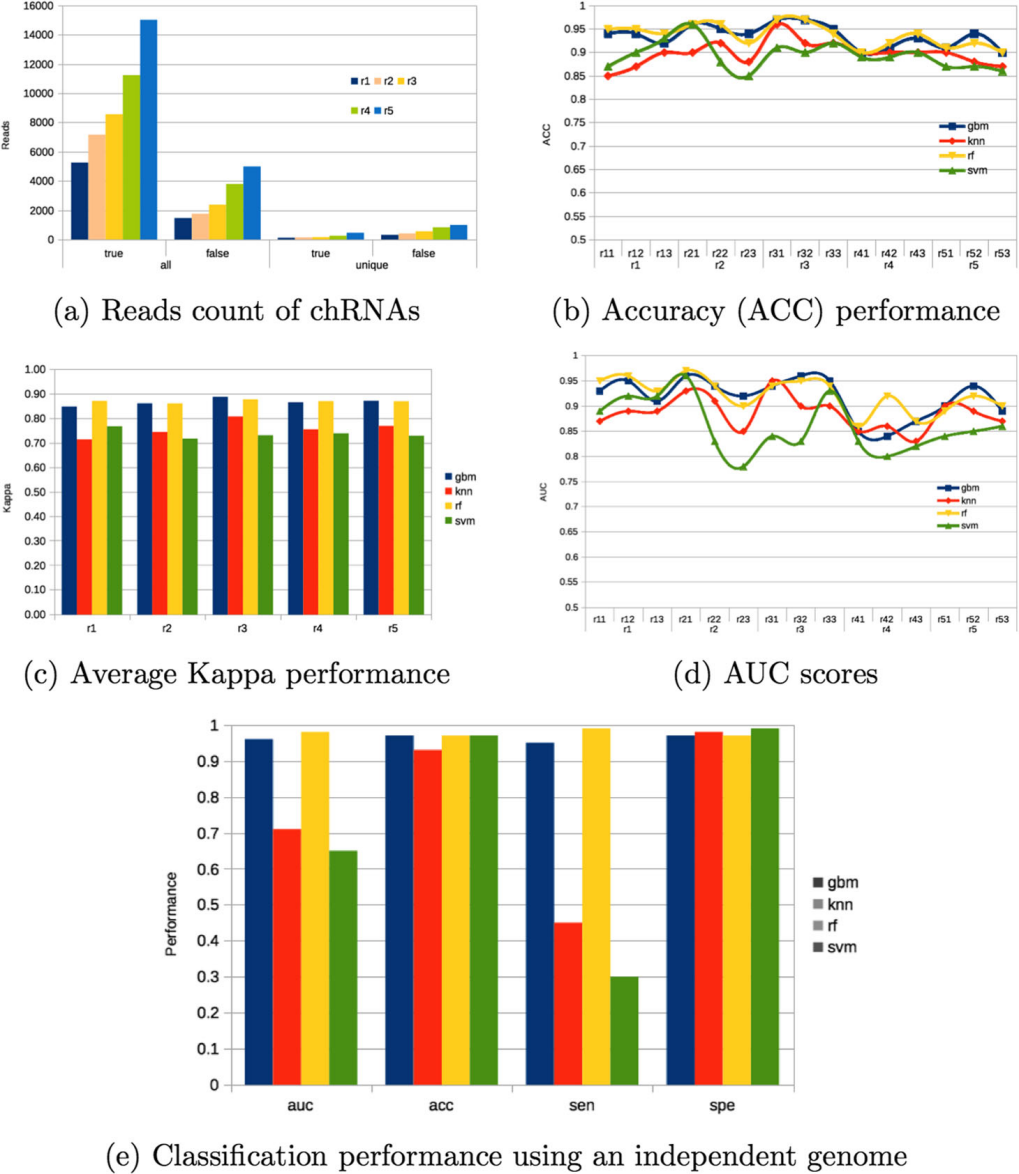
Overall performance of several chRNA classifiers at the read level. Progressive sampling using five genomes along chRNA mutation profiles, from “r1” to “r5”. Training were performed with a 10-fold cross validation scheme along with a tuning grid to each ML technique. Additionally, we repeated each run three times (e.g., rX1, rX2, and rX3) to check models stability. Thus, having a total of 15 performance points. Models’ performance were average to each run (r1 to r5). The chRNA’s classes distribution along reads (**a**). Performance metrics of ACC (**b**), Kappa (**c**), and AUC (**d**) highlight the robustness of ensemble models. Classification performance using another independent genome (**e**)

A total of five *Human* (GRCh38 assembly) mutated genomes were generated. They were used in a progressive sampling scheme for benchmarking several supervised classifiers. For each mutated genome, 40 millions of paired-end reads with read length of 100bp were generated. CRAC selected all reads and labelled them according to their associated class. Next, five features emerging from CRAC’s algorithm were extracted as follows: i) score_break_length, it describes the size of the break (adjacent unmapped k-mers), particularly those presenting sizes higher than the average; ii) score_nb_merge, it evaluates if the break corresponding to the chimera has a number of merges < to the related parameter; iii) score_break_variance, it checks the inter-quartile range of support values in the break; iv) score_is_duplicate, it checks if the chimera is ambiguous; v) score_has_repeat, it evaluates if the break is in a repeated region. Given that several chRNAs reads can map to one particular chimeric event, some of those reads may hold the same values for the previously described set of features. Therefore, only unique reads per chRNAs are taken into account in the model building phase. Table 1 summarises the chimeric reads distribution within the progressive sampling runs. The first run (r1) has one transcriptome and, progressively, one more were added to the sample up to having all five mutated transcriptomes in the fifth run (r5) (Fig. 2 a).

**Table 1:** The chRNAs reads distribution within the progressive sampling

	(A)ll reads		(U)nique reads		% U/A	
	True	False	True	False	T	F
r1	5253	1462	118	316	2.25	21.6
r2	7169	1761	145	415	2.02	23..6
r3	8563	2377	163	560	1.90	23.6
r4	11238	3795	259	836	2.30	22.03
r5	15016	4988	460	995	3.06	19.95

### Supervised learning strategies

The challenge of finding chRNAs can be seen as a binary classification problem (positive class: true chimera and negative class: false chimera) [12]. Once having the mutated genomes we can move towards the evaluation of several supervised learning strategies for mining chimeras. The comparison study takes into account distinct supervised learning strategies, namely, one lazy learner (KNN: K-Nearest Neighbors), one eager-learner (SVM: Support Vector Machines) and two ensemble learners (RF: Random Forest and GBM: Grandient Boosting Machines). Each ML strategy has a particular way to generalize the search space.

Nearest-neighbor classifiers are based on learning by analogy, by comparing a given test instance with training instances that are similar to it. Support Vector Machines uses a linear model to implement nonlinear class boundaries. SVM transform the input using a nonlinear mapping, thus, turning the instance space into a new space. Random forest is a well-known ensemble approach for classification tasks proposed by Breiman. Its basis comes from the combination of tree-structured classifiers with the randomness and robustness provided by bagging and random feature selection. Gradient boosting machines consecutively fits new models to provide more accurate estimate of the response variable. Thus, new base learners are built being maximally correlated with the negative gradient of the loss function associated with the entire ensemble. The loss function applied can be arbitrary, so as an example, if the error function is squared-error loss, the learning procedure would result in consecutive error fitting.

### Building models

Model building and evaluation was carried out with the caret R package [16]. We used the built-in *t* *u* *n* *e*() function for resampling, tuning and optimisation of all selected classifiers. We have also adopted a progressive sampling strategy to better understand the impacts of adding more observations to the classifiers performance. We first started using one simulated data set (incorporating a mutation process), and next more data were added sequentially up to having all five genomes. To each run we have set aside one third of the data for the test phase while the remaining two third were used for model building. Training were performed with a 10-fold cross validation scheme along with a tuning grid to each ML technique. Additionally, given that the data split (training/test) were random, we repeated each run three times to check models stability, providing fifteen performance points. As an example r11, r12, and r13 are subsets of the r1 data set (Fig. 2 b and d). Models’ performance were average to each run (Fig. 2 c).

## Results

### Towards a classification baseline at the “read” level

Give the absence of a classification baseline for the problem of finding chRNAs, let us assume, essentially, the performance results we got from the first run (r1) of KNN as the initial baseline. The baseline accuracy is also known as the null rate. In machine learning it is also a good practice to start with simple classifiers rather than sophisticated ones [9]. The KNN provided average AUC performance of 88 % (ACC=87 %, Kappa=0.71). Kappa measures how closely the instances labeled by the classifiers matched the data labeled as ground truth, controling for the ACC of a random classifier as measured by the expected accuracy. Thus, the Kappa for one classifier is properly comparable to others Kappa’s classifiers for the same classification problem. We will make use of Kappa as the unbiased metric to compare the selected classifiers along all the progressive sampling strategy.

The general idea behind of a progressive sampling is straightforward. We start with some chRNA reads arising from a individual genome and then, iteratively, build a model, evaluate its performance, and acquire additional chRNA reads from another individual genome. Initially, we were planing to use a simple stop criteria along sampling until getting a performance plateau. Thus, we might be able to stop having more data in the third run (Fig. 2 b). However, we decided to have two more additional runs, and, as it can be observed, all models loosed generalization power in the fourth run. All simulations have the same genome simulator parameters and mutation profile and expression profiles, though “randomness” plays quite distinctly to the fourth ones. It might be partially due to the fact that the associated data sets are unbalanced but, every run has about the same proportion of true and false chRNAs (see Table 1). Another hypothesis is that, perhaps, mutations in the fourth genome is biased to patterns in alternative splicing.

**Fig. 3:**
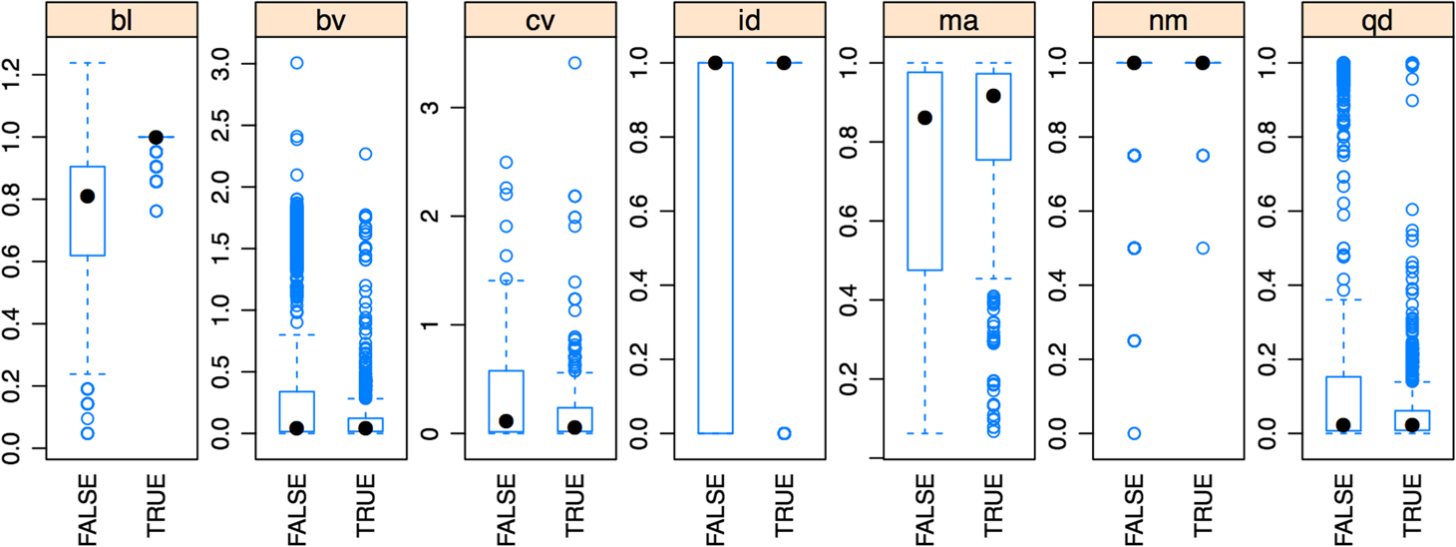
Box-plots of the selected features to discriminate chRNAs (TRUE and FALSE) at the read level. From left to right (score_break_length, score_break_variance, coefficient_variation, score_is_duplicate, mean_amplitude, score_nb_merge, coefficient_dispersion_interquartile). Best discrimination results are obtaining with the features score_is_duplicate, coefficient_variation, and score_break_length

Ensemble learning strategies were more sensitive to this “random perturbation” added by the fourth genome. The feature score_has_repeat does not bring any discriminative power, being eliminated from the set of predictors. Additionally, we have worked on improving performance of classifiers by introducing three more features, enhancing the break profile (Fig. 3), namely: i) coefficient_variation, the coefficient of variation of the support of the k-mers over the break; ii) mean_amplitude, mean amplitude of the break support; and iii) coefficient_dispersion_interquartile, *Q*3−*Q*1/*Q*3+*Q*1. Finally, the best performance results were obtaining by combining the features score_is_duplicate, coefficient_variation, and score_break_length (see Table 2).

**Table 2:** The average performance of classifiers at the read level using the top-3 more discriminative features (score_break_length, coefficient_variation, score_is_duplicate)

	GBM	RF	KNN	SVM
Acc	95.01	95.16	94.29	93.49
Spe	92.64	93.02	92.44	92.05
Sen	96.42	96.42	95.39	94.35
Kappa	89.31	89.62	87.79	86.13
AUC	94.53	94.72	93.91	93.20

It is important to mention that a grid of optimisation were applied to all supervised classifiers. Surprisingly, the SVM provided one of the most unstable results, even when compared to KNN. We hypothesise that the search space might be a set of Boolean functions, and given that both RF and GBM employ tree stumps, random perturbations are handled efficiently by ensemble of Booleans rules generated by these classifiers. Thus, in this particular study, indeed, a more powerful learner is better than a less powerful one. Ensemble learning strategies demonstrated to be more robust to this classification problem, providing an average AUC performance of 95 % (ACC=94 %, Kappa=0.87 %) (Fig. 2).

### Towards a classification baseline at the “event” level

**Fig. 4:**
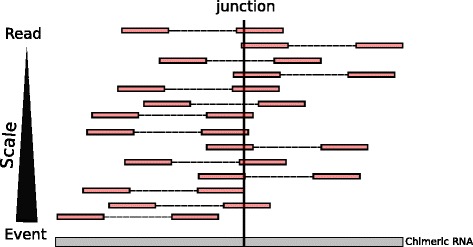
The chRNA granularity. Scaling chRNAs classification from read to event level. The event level is basically the aggregation of all reads corresponding the same chimeric junction at read level and its associated functional (biological) annotation

The problem of chRNAs classification can also be tackled from the perspective of the event level. The event level is basically the aggregation of all reads corresponding the same chimeric junction and its associated functional (biological) annotation (Fig. 4). Gingeras et al. [1] define chRNAs events (classes) as follows: i) individual RNAs can be transcribed on separate chromosomes (Class 1), on the same chromosome but with a different genomic order from that found in the mature RNA (Class 2), on the same chromosome but transcribed from different strands (Class 3), or on the same chromosome but from different alleles (Class 4). Before grouping chimeric reads into an event, it is required a proper filtering stage of potential methodological artifacts at the read level. In the previous section we have conducted a first attempt at classifying chRNAs at the read level. These results allowed us to refine the current chimera score on CRAC (CracScore) eliminating false positives that might strongly bias the classification of chRNAs at the event level. This score was based on a set of selected significant rules extracted from the Random Forest model, by the utilization of the *inTree R package*.

Once having applied CRAC with an “optimal” score, one can go forward to the aggregation and annotation of chRNAs at the event level. We use the *cractools extract* command from the *CracTools* (http://cractools.gforge.inria.fr/) to aggregate chRNAs. Then, we annotate chRNAs and use it as filter to reclassify those involving the same gene as splicing or alternative splicing and also pseudogene as artefact. Without loss of generality, we continue to tackle the chRNAs prediction at the event level as a binary classification problem. We also make use of the terms “filtered aggregation” to differentiate this approach from CRAC. We have implemented and test this tool along with a fixed value of 0.7 on a new simulated data set. This data set follows the same strategy as discussed in previous section, so 40 millions of paired-end reads with read length of 100bp were generated. This simulation also covers 180 chromosome translocation over *Human* (GRCh38) genome. Flux Simulator were also used to express 121 translocation over 46938 reads. The following table summarizes the analysis (Table 3).

**Table 3:** Classifiers performance at both read and event level

	Total	True	False	Accuracy	Sensitivity
read level (CRAC)	5 444	4 964	480	91.18	10.58
Filtered aggregation	153	83	70	54.25	68.60

The performance of the classification at the event level, as it might be observed in (Table 3), is not better than a “random guess”. In fact, this is mainly due to the fact that i) aggregation does not take into account a customized classification model to discriminate chimeric events, and ii) chimeric (false positives) reads continue passing through the filters (CracScore), causing trouble to the classification at the event level. Therefore, it is advisable to employ a stacking model, so chimeric reads that are not properly filtered out at the read level, can be, with the advantages of having additional functional information, be enhanced to engineering new features playing interesting role at building a model at the event level.

In order to provide a classification baseline at the event level, we carried out new simulations, avoiding the bias that might be associated while evaluating classification performance at both levels using the same data. In fact, it opens the possibility to explore new covariates, specifically suitable for chRNA predictions at the event level. We have generated six data sets (3 RNA-seq 100bp + 3 RNA-seq 200 bp), corresponding to 1634 chimeric events (1257 = False and 377 =True). A feature set of eleven covariates were carefully designed, though, without loss of generality, only the three most discriminative ones are described: i) coefficient_variation, false chimeric events present higher variability within the junction support than true ones. This is partially due to false localisation inferred wrongly by repetitive regions; ii) fusion_distance, the larger the fusion distance is, the less is the likelihood of the event be a false positive; iii) junction_support, once the support is low one might expect of being a false event, give that the junction support is covered by a few or none reads.

The classification baseline at the event level explores the same strategy discussed in the “building model” section. We also provided to each model the average and standard deviation of the three repetition of 10-fold cross validation (Table 4).

**Table 4:** The overall performance of classifiers at the event level using the top-3 more discriminative features (coeficient_variation, fusion_distance, juntion_support)

	RF		GBM		KNN		SVM	
	mean	sd	mean	sd	mean	sd	mean	sd
ACC	97,98	0,12	97,85	0,20	97,43	0,61	97,98	0,12
Spe	95,67	0,84	95,39	1,02	94,63	2,04	95,94	0,65
Sen	98,71	0,11	98,77	0,13	98,33	0,23	98,63	0,25
Kappa	94,46	0,34	94,09	0,66	92,92	1,72	94,45	0,50
AUC	97,01	0,48	96,65	0,23	96,47	1,10	97,29	0,30

### The fidelity of supervised classifiers in the prediction of chRNAs

We have performed an evaluation using another *Human* mutated genome, as an independent test set, and the results again are promising for ensemble learners as RF and GBM. We again use our genome simulator, and a total of 80 millions of paired-end reads with read length of 100bp were generated for this new mutated genome. For this last evaluation, we have selected the respective models for each classifier obtained in sampling r5 to predict chRNAs within this new genome (Fig. 2 e). Despite small performance differences across ensembles, the RF provided the best overall results (AUC=98 %) (Fig. 2 e).

We carried out another evaluation using a generated mutated *Drosophila* genome (40M reads having 100bp) to check whether the *Human*-based models could predict efficiently chRNAs in other, let us say, distant organism. As it was expected no classifier were better than a random guessing. Such observation is due to the fact both organisms have a distinct “chimeric profiles” with respect to the number of chromosomes, the arrangements of the exons and evolutionary rate of mutations. Therefore, classification margins boundaries built for *Human* cannot hold for *Drosophila*.

**Fig. 5:**
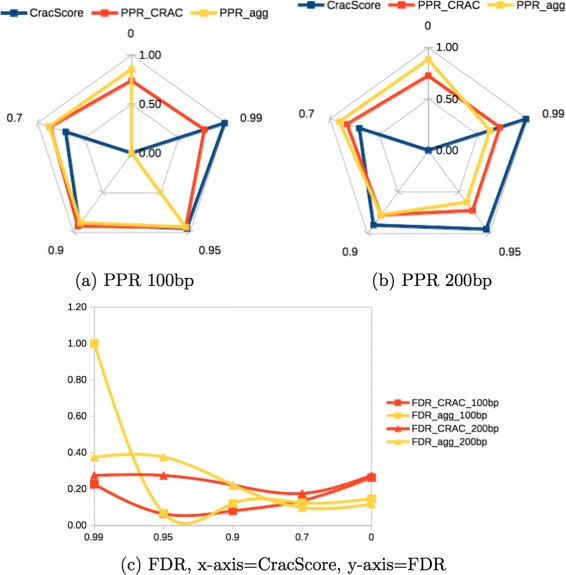
The fidelity of chRNA classifiers using simulated RNA-seq data. The radar chart of using distinct chimera scores (0.7, 0.9, 0.95, 0.99, and 1) on CRAC (CracScore) and its impact on prediction performance. CracScore to 0.7 is a good compromising between precision (PPR) 100bp (**a**), PPR 200bp (**b**), and false discovery rate (FDR) (**c**). Moreover, the higher the CracScore is the higher is the precision though with the cost of increasing the FDR at the read level

It is remarkable that classifiers employing an ensemble learning strategy are most suitable for the challenge of prediction chRNAs. Furthermore, a stacking model where false chimeric reads are filtered out at the read level, being followed by a classifier at the event level (embedding functional e biological covariates) is strongly recommended. We have generated two more RNA-seq data sets covering 40M reads (1x100 bp and 1x200 bp) to evaluate the strength of the prediction in both levels (Tables 5 and 6). The first genome has 121 chRNA events (translocations quantified along 46936 reads), and the latter has 115 events (translocations quantified along 101093 reads). We observe from (Fig. 5) that setting a CracScore to 0.7 is a good compromising between precision (PPR) and false discovery rate (FDR). Moreover, the higher the CracScore is the higher is the precision. However, such precision come with the cost of increasing the FDR at the read level, being compensate by lower FDR values at the event level. The size of the read does not imply in having better classification performance at both levels but, can increase the chance of having more reads covering chimeric junction, and consequently long reads, can help in boosting prediction at the event level.

**Table 5:** Performance of classifiers at both read and event levels using 100 bp

Data	Simulated RNA-Seq 40M reads 100 pb									
CracScore	0		0.7		0.9		0.95		0.99	
Class	True	False	True	False	True	False	True	False	True	False
Count reads	4196	1498	3815	600	2644	226	2056	139	477	139
Aggregation	56	209	56	89	29	31	16	8	2	8
Filtered aggregation	35	6	35	5	22	3	14	1	0	1

**Table 6:** Performance of classifiers at both read and event levels using 200 bp

Data	Simulated RNA-Seq 40M reads 200 pb									
CracScore	0		0.7		0.9		0.95		0.99	
Class	True	False	True	False	True	False	True	False	True	False
Count reads	13164	4965	10238	2178	7099	2015	4732	1789	4732	1789
Aggregation	57	266	55	107	28	44	12	25	12	25
Filtered aggregation	38	5	37	4	14	4	5	3	5	3

**Fig. 6:**
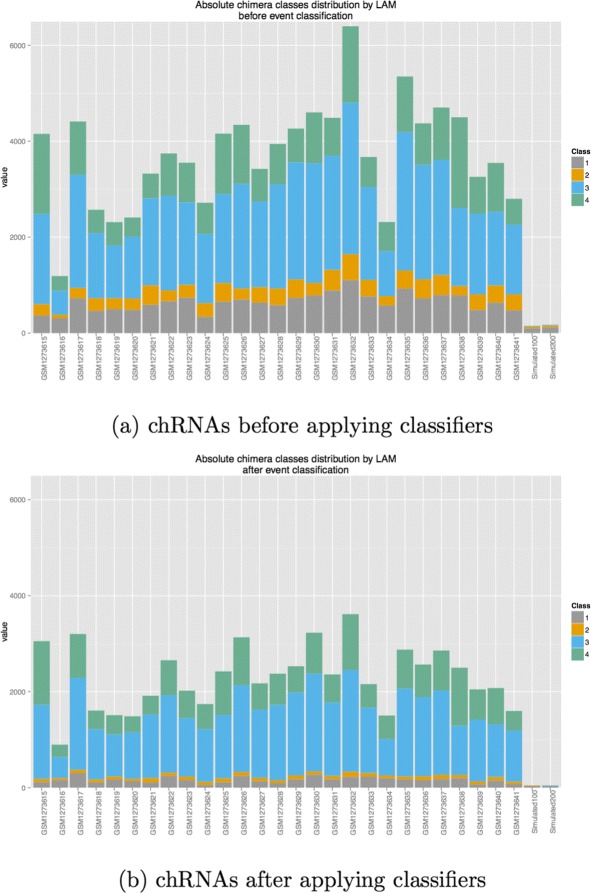
The fidelity of chRNA classifiers using real RNA-seq data. The x-axis are related to samples, while the y-axis presents the number of chimeric events. The same amount of sequence artefacts found in simulated data can be equally distributed on real data (**a**). The classification results obtained after applying the pipeline highlight a significant reduction in chRNA class distribution (**b**)

To conclude our experimental study, we have applied the proposed pipeline for the prediction of chRNAs in real RNA-seq data (Illumina HiSeq 2000 *Homo sapiens*) from a set of twenty-seven patients having acute myeloid leukemia. The goals of this study are to obtain a comprehensive study of mutations and gene expression in human acute myeloid leukemia (AML). All sequence data are freely available at Gene Expression Ominibus (GEO) database within the accession number GSE49642. From the previous simulation study, we observed that most of the false chRNA events belong to the “Class_1” (60 %), while the remaining false events are shared between “Class_2”(30 %) and, “Class_3” and “Class_4” together (10 %). We hypothesise that the same amount of sequence artefacts found in simulated data can be equally distributed on real data. Thus, it is expected that classifiers might be able to eliminate successfully many of these false events. Indeed, the classification results obtained after applying the pipeline highlight a significant reduction in chRNA class distribution (Fig. 6). Biological process that generates Class_1 events are well-known. However, the main part of chRNA found in real data belong to Class_3 and Class_4. These chRNA have various mecanisms that are still little known. The simulation must be continuously improved to integrates new biological mecanisms but also other properties as cellular heterogeneity and library conception bias.

The proposed benchmark highlights the fidelity of supervised classifiers in the problem of chRNAs prediction. Though, it is always important performing biological validation of these events to confirm the biolo gical soundness of all discovered chRNAs.

## Conclusion

We proposed a modelization strategy that can be used to increase the tools performances in context of chRNA classification. We improve the models dynamically using a simulated data generator, called SimCT (not yet published), that permit to continuously integrate new complex chimeric events.

We have developed a benchmark pipeline incorporating a mutation genome process and simulated RNA-seq data to evaluate the fidelity of using classifiers to predict chRNAs. The simulated sequencing reads were aligned and annotated by CRAC. CRAC analyzes the RNA-seq data, integrating genomic location and local coverage, allowing biological predictions in one single step which is not available in any other fusion finder tools. Moreover, we enhance chRNAs classification by building models at both levels of read and event. Aggregating reads at the event level allows us to incorporate biological covariates and improving models’ generalization along with the presented experimental study. We envisage focusing on developing models and improving biological annotation at event level which will be addressed in a future work.

Simulated and real RNA-seq data were carefully designed to explore feature engineering and model performance of several classifiers, unraveling the role of ensemble learning strategies in finding chRNAs. As far as we are concerned the present study provides the first common benchmark data, as well as classification baseline for the identification of chRNAs in both levels of reads and events.

## Additional file

Additional file 1
**Figure S1**. Anatomy of a chimeric junction identified with CRAC algorithm and its potential extracted features for machine learning benchmarking. (PDF 41.8 kb)

## Abbreviations

AML: Acute myeloid leukemia
AUC: Area under the curve
chRNA: Chimeric RNA
GEO: Gene expression Ominibus
Kappa: Cohen’s kappa coefficient
SNV: Single nucleotide variants
