# Supplementary material for: On the evaluation of the fidelity of supervised classifiers in the prediction of chimeric RNAs
Source: BioData Min. 2016 Nov 2;9:34. doi: 10.1186/s13040-016-0112-6 (PMC5090896; doi:10.1186/s13040-016-0112-6)
Supplement: Additional file 1 — Figure S1. Anatomy of a chimeric junction identified with CRAC algorithm and its potential extracted features for machine learning benchmarking. (PDF 41.8 kb) [file 13040_2016_112_MOESM1_ESM.pdf]

Anatomy of a chimeric junction identified with CRAC algorithm

Input data

Chimeric event

Chromosome A and B are translocated together. A chimeric transcript is produced from the fusion of two genes, one from chromosome A and the other from chromosome B.

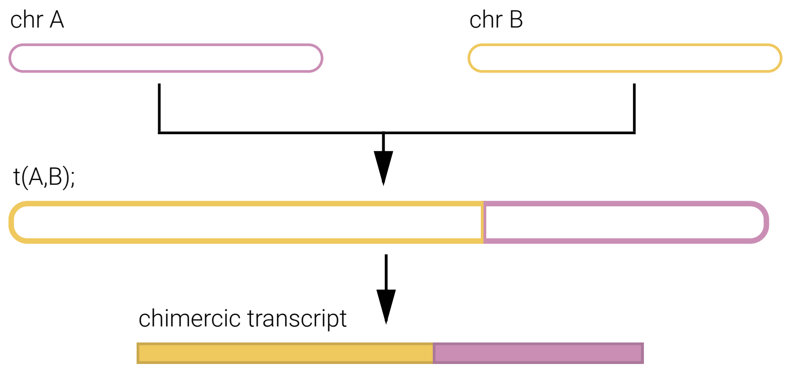

chimeric read

A read overlapping the chimeric junction.

ATGCTGTAGGGCTGATCGAC

CRAC algorithm

CRAC k-mer analysis

CRAC procedure to analyse reads relies on the analysis of k-mer profiles.

```
1   A T G C T G
2     T G C T G T
3       G C T G T A
4         C T G T A G
5           T G T A G G
6             G T A G G G
7               T A G G G C
8                 A G G G C T
9                   G G G C T G
10                    G G C T G A
11                      G C T G A T
12                        C T G A T C
13                          T G A T C G
14                            G A T C G A
15                              A T C G A C
```

break
Def: interruption of k-mer localization.
One false positive location in green.

localization profile

Number of time each k-mer is found on the reference genome.

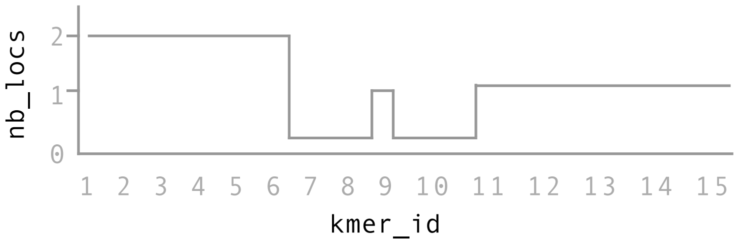

support profile

Number of time each k-mer is found in the input dataset.

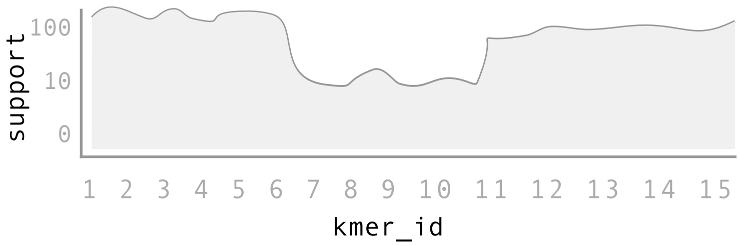

ML features

score break length

Measure of distance between the observed break length and the theoretical break length.

score_break_length = observed_break_length() / (k - 1) = 1

score is duplicated

If the k-mer location around the break is ambiguous.

score_is_duplicate = true

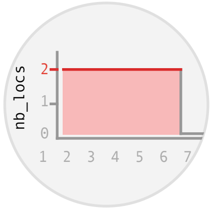

score coefficient variation

Coefficient variation of the support profile.

score_coeff_var = std_dev(support[]) / mean(support[])
